# Reply to Itoh et al., “Potential Use of Iron-Limiting Therapy against Cryptococcus neoformans and Effects of Caspofungin on the Host Immune System”

**DOI:** 10.1128/msphere.00379-22

**Published:** 2022-08-22

**Authors:** Maureen J. Donlin

**Affiliations:** a Edward A. Doisy Department of Biochemistry and Molecular Biology, Saint Louis University School of Medicine, St. Louis, Missouri, USA; University of Georgia

**Keywords:** iron chelators

## REPLY

We appreciate the concerns raised by Ito et al. on the clinical implications of therapies targeting iron transport or membrane permeability in combination with caspofungin. The focus of our work was to describe the basic biological mechanisms of the resistance of Cryptococcus neoformans to caspofungin and not to make recommendations for clinical therapy. However, understanding the basic mechanisms can be the basis for development of therapeutics, which can involve very complex interactions.

We observed only moderate sensitivity of *cft1Δ* to caspofungin, implying that iron transport mediated by Cft1 does not play a significant role in caspofungin resistance, under the conditions tested. It does not rule out a possible synergistic role for Cft1 and Cft2 in iron uptake. Furthermore, we did not directly test *cft1Δ* sensitivity to caspofungin under host-mimicking conditions and thus cannot rule out synergistic effects with iron chelation *in vivo*.

In terms of membrane integrity, we conclude, “In the future, the most likely path to new therapies will be the combination of caspofungin with inhibitors that can significantly alter the composition or morphology of the cryptococcal cell wall or membrane.” This conclusion is reasonable, given that our data clearly show that altered membrane composition sensitizes C. neoformans to caspofungin while potentially interfering with caspofungin accessibility to yeast cells. These potential compounds must, of course, be specific to fungal enzymes and not interfere with host immunity or membranes.

